# Classification and grading of canine malignant mammary tumors

**Published:** 2013

**Authors:** Abbas Tavasoly, Hannaneh Golshahi, Annahita Rezaie, Mohammad Farhadi

**Affiliations:** 1 *Department of Pathology, Faculty of Veterinary Medicine, University of Tehran, Tehran, Iran; *; 2 * Department of Pathobiology, Faculty of Veterinary Medicine, University of Shahid Chamran, Ahvaz, Iran; *; 3 *Department of Hematology, Iranian Blood Transfusion Organization, Tehran, Iran.*

**Keywords:** Classification, Dog, Grading, Mammary tumor

## Abstract

Histological grading is a good parameter to stratify tumors according to their biological aggressiveness. The Elston and Ellis grading method in humans, invasive ductal breast carcinomas and other invasive tumors are routinely used. The aims of this study were classification of mammary gland tumors and also application of a human grading method in canine mammary carcinoma. The samples included 37 tumors of mammary glands. Mammary tumors were carcinomas (n = 32) and sarcomas (n = 5). The carcinomas were classified as simple carcinoma 56.8% (n = 21), complex carcinoma 13.5% (n = 5), carcinoma arising from benign tumor 10.8% (n= 4) and special type of carcinoma 5.4% (n = 2). Out of 32 carcinomas studied, 37.5% (n = 12) grade I, 46.9% (n = 15) grade II and 15.6% (n = 5) grade III. This study demonstrated that the Elston and Ellis method of histological grading in canine mammary tumor is a reliable prognostic factor which is correlated with histopathological classification.

## Introduction

Canine mammary gland tumors (CMGTs) are one of the most common neoplasms of bitches.^1^^-^^3^ Most frequently mammary gland tumors are found in 5 years and older bitches.^4^ Dachshunds, cocker spaniels, toy poodles, German shepherds, mixed – breed dogs have been reported to have an increased incidence of mammary neoplasia.^5^ Mammary gland carcinomas are quite heterogeneous in terms of morphology and biological behavior,^2^ and have been the focus of intensive research over the last few decades. Simple and complex carcinomas are recorded as the most common type of malignant CMGTs.^6^ Canine mammary sarcomas often have multi-differentiation (bone, cartilage, and fat) which is not unusual in human mammary sarcomas. Sarcomas resembling (malignant) cystosarcoma phyllodes in women appear to be very rare in the dog. There is need for further studies on the histogenesis and biological behavior of mammary sarcomas.^7^

Domestic pets are particularly valuable models, as possible sentinels for human environmental and life-style risks. Experimental models, principally genetically engineered mice (GEMS), have been useful for investigating the role of steroid hormones, hormone receptors, and other growth factors in the pathogenesis of breast cancer. However, rodents differ considerably in mammary gland development and types of breast cancer from women. Furthermore, many mammary gland cancers in mice are viral- or toxin- induced, so the validity of these models has been questioned. The high prevalence of spontaneous mammary cancer in domestic dogs and cats closely mimics the disease in women, making these species more suitable comparative models.^8^

Histological grading is a good parameter to stratify tumors according to their biological aggressiveness.^9^^,^^10^ The Elston and Ellis grading method in humans, invasive ductal breast carcinomas and other invasive tumors are routinely graded.^11^ The “Elston and Ellis method” is the most common histological grading method for invasive carcinomas and shows a strong correlation with prognosis.^11^^,^^12^

Surgery remains the basic treatment for dogs and cats with most type of mammary gland tumors. The exceptions are inoperable disease (e.g., inflammatory carcinoma of the dogs) and distant (organ) metastasis. One of the major problems in veterinary oncology is accurate prognosis for post-surgical mammary cancer cases. In human breast cancer and in canine mammary tumor, histological type, histological grade and lymph node involvement are standard prognostic features.^2^^,^^11^^-^^13^ Morphological criteria alone may be insufficient for a proper diagnosis because when only histologically determined, benign tumors may incidentally give rise to metastasis. Despite benign biological behavior, canine complex adenomas and mixed tumors often show histomorphological evidence of malignancy (carcinoma or sarcoma in benign tumor).^14^ Tumor grade and degree of invasion (stage) are also of prognostic signiﬁcance.^13^

The aim of this study was classification and also application of a human grading method in canine mammary carcinoma. The incidence of canine mammary gland carcinoma is common in Iran, however, only Rezaie *et al.,* used this method to grade these tumors.^15^

## Materials and Methods

**Specimens. **Thirty seven spontaneous tumors of the mammary gland from bitches aged from 4 to 15 years (average 8.5 years), of various pure or mixed breed during September 2009 to September 2011 for diagnostic purposes were obtained. They were selected from cases treated surgically or from archive of Pathology Department of Faculty of Veterinary Medicine, University of Tehran, Tehran, Iran. In surgically removed samples, the mammary tumors were excised by simple mastectomy or regional mastectomy, with or without the superﬁcial inguinal lymph nodes. Tissues, submitted in 10% neutral buffered formalin were embedded in paraffin wax. Sections (5 µm thick) were cut and routinely stained with hematoxylin and eosin (H & E) for histological examination. 

**Histopathological evaluation. **Tumors were classiﬁed according to the World Health Organization (WHO) criteria for canine mammary lesions^6 ^based on the most pronounced histological pattern observed in more than 50.0% of the tumor mass. Whenever tumors displayed multiple morphological patterns, without more prominent growth pattern present in 50.0% of the tumor mass, lesions were classiﬁed as tumors with mixed morphology tumor.

**Tumor grade. **Grade was determined according to the Elston and Ellis scoring system^12^ based on the assessment of three morphological features: (1) the degree of glandular differentiation assessed by tubular formation; (2) nuclear pleomorphism, and (3) mitotic activity. Each parameter was graded into three categories, to which a score of 1-3 was assigned as follows: (1) tubule formation (tumor had more than 75% tubules = 1, 10-75% of tumor had tubule formation = 2, and < 10% tubules; when evaluating tubules, only structures exhibiting clear central lumina were counted = 3); (2) nuclear pleomorphism (small regular uni-form cells = 1, moderate nuclear size and variation = 2, and marked nuclear variation = 3), and (3) number of mitosis per 10 high power ﬁelds (HPF; 40 objective lens, ﬁeld area 0.239 mm^2^) (0-7 mitosis per 10 HPF = 1, 8-16 mitosis per 10 HPF = 2, and ≥17 mitosis per 10 HPF = 3). The scores of all three components were added together to give a total of 3-9 points. Grade was allocated by an arbitrary division as follows: (1) grade I, well differentiated or low grade: 3-5 points; (2) grade II, moderately differentiated or intermediate grade: 6-7 points, and (3) grade III, poorly differentiated or high grade: 8-9 points (Table 1). Both classification and grading were independently examined by two observers and when there was a divergence of opinion, an agreed diagnosis was reached by using a multi-headed microscope.

**Table 1:** Summary of semi-quantitative methods for assessing histological grades in mammary carcinomas as proposed by Elston and Ellis (1998).

**Features**	**Score**
**Tubule formation**	
˃ 75%	1
10-75%	2
˂ 10%	3
**Nuclear pleomorphism**	
Small regular uniform cells	1
Moderate nuclear size and variation	2
Marked nuclear variation	3
**Mitotic counts** *	
0-7	1
8-16	2
≥ 173	3
**Olympus BX-40 Microscope**	
Objective	40×
Field diameter (mm)	0.55
Field area (mm^2^)	0.239

* Number of mitosis per 10 fields at the tumor periphery.

## Results

The samples under evaluation included 37 tumors of mammary glands. Results showed that all samples were malignant 100% (n = 37) and 86.5% (n = 32) and 13.5% (n = 5) of mammary tumors were carcinomas and sarcomas, respectively. The most frequently represented tumor type were simple carcinoma 56.8% (n = 21) followed by complex carcinoma 13.5% (n = 5) sarcoma 13.5% (n = 5) carcinoma arising from benign tumor 10.8% (n = 4) and special type of carcinoma 5.4% (n = 2). 

**Fig. 1:**
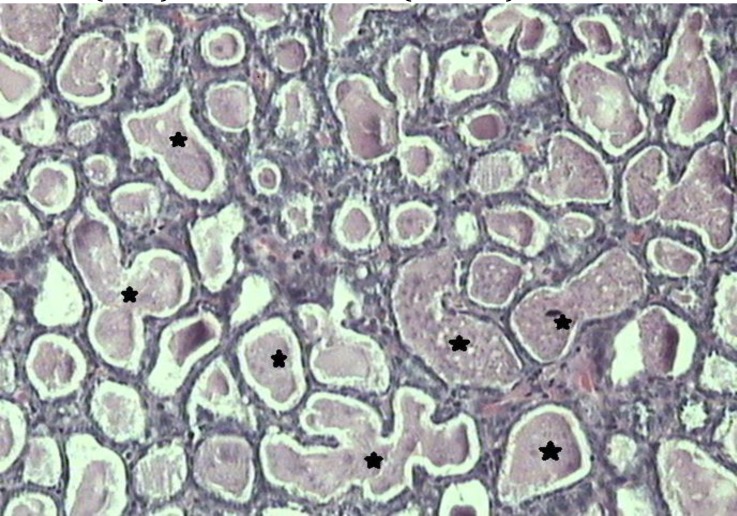
Tubulopapillary carcinoma (grade 1). Note the many tubule formations with clear lumen (stars), (H & E, 100×).

**Fig. 2:**
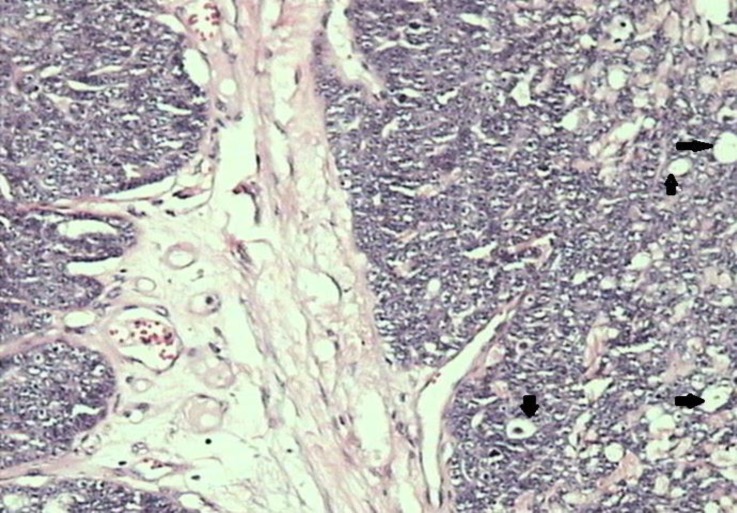
Cribriform carcinoma (grade 2). Note the neoplastic epithelial cells forming a sieve like arrangement (arrows), (H & E, 100×).

**Fig. 3:**
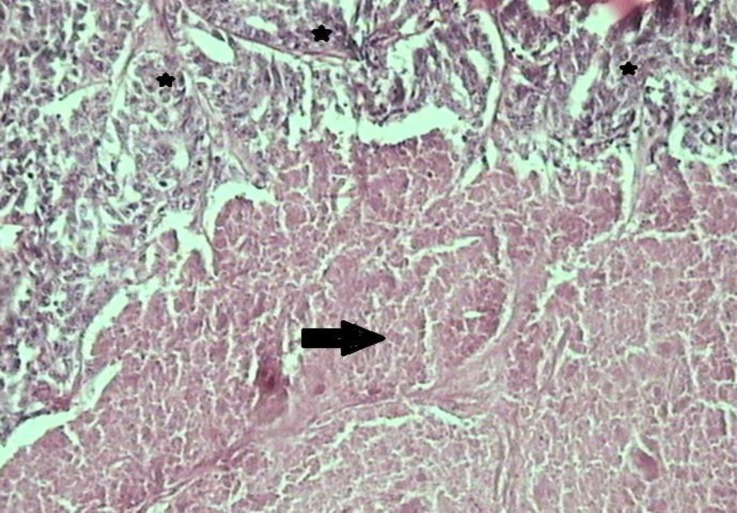
Solid carcinoma (grade 3). Note the cells which are predominantly arranged in solid sheets, cords, or masses, without lumina (stars) and extensive coagulative necrosis (arrow), (H&E, 100×).

Simple carcinomas (n = 21) were sub classified to 80.9% (n = 17) tubulopapillary carcinoma, 4.8% (n = 1) solid carcinoma and 14.3% (n =3) cribriform carcinoma (Figs. 1, 2 and 3). Special type of carcinomas (n = 2) were sub classified to 50.0% (n= 1) lipid-rich tumor and 50.0% (n = 1) spindle cell tumor. Sarcomas (n = 5) were sub classified to 80.0% (n = 4) carsinosarcoma (Figs. 4a, 4b and 5) and 20.0% (n = 1) chondrosarcoma (Table 2).

**Table 2:** Frequency of type of 37canine mammary tumors.

**Histological type**	**Number**
**Carcinomas**	32
Simple carcinoma	21
Tubulopapillary carcinoma	17
Solid carcinoma	1
Cribriform carcinoma	3
Complex carcinoma	5
Special type of carcinoma	2
Lipid-rich carcinoma	1
Spindle cell carcinoma	1
Carcinoma arising in benign tumor	4
**Sarcomas**	5
Carcinosarcoma	4
Chondrosarcoma	1

The carcinomas of this study were as follow: Out of 32 carcinomas studied, 37.5% (n = 12) grade I, 46.9% (n = 15) grade II and 15.6% (n = 5) grade III with high mitotic index (Fig. 6). Tumors were graded I included 10 simple carcinoma (all were tubulopapillary carcinoma) and two carcinoma arising in benign tumor. Grade II samples consisted of nine simple carcinoma (seven tubulopapillary carcinoma and one cribriform carcinoma), five complex carcinoma and one carcinoma arising in benign tumor. In grade III group, two simple carcinoma (one solid carcinoma and one cribriform carcinoma), one carcinoma arising in benign tumor and two special type of carcinoma (one lipid-rich tumor and one spindle cell tumor) existed. In the present study, there was an evident correlation between histological type and grade (Tables 3, 4 and 5).

**Fig. 4a:**
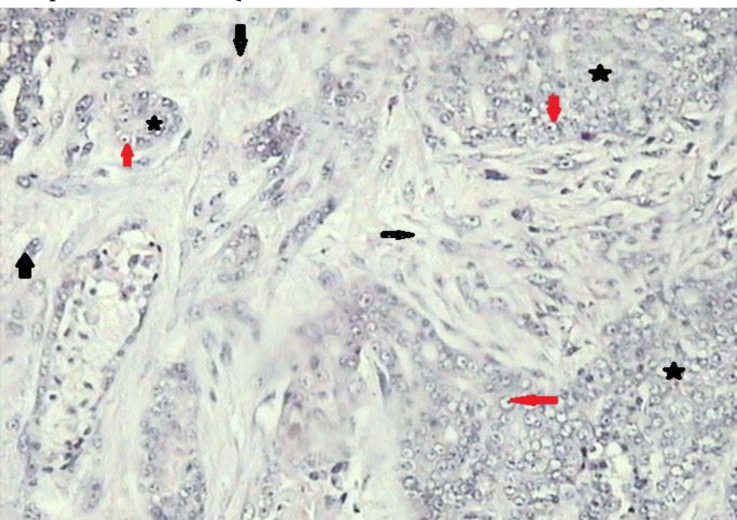
Carcinosarcoma. Note to the neoplasttic epithelial cells arranged in masses to form solid pattern (stars), vesicular nuclei with prominent nucleoli (red arrows) and cartilage differentiations (black arrows), (H & E, 100×).

**Fig. 4b:**
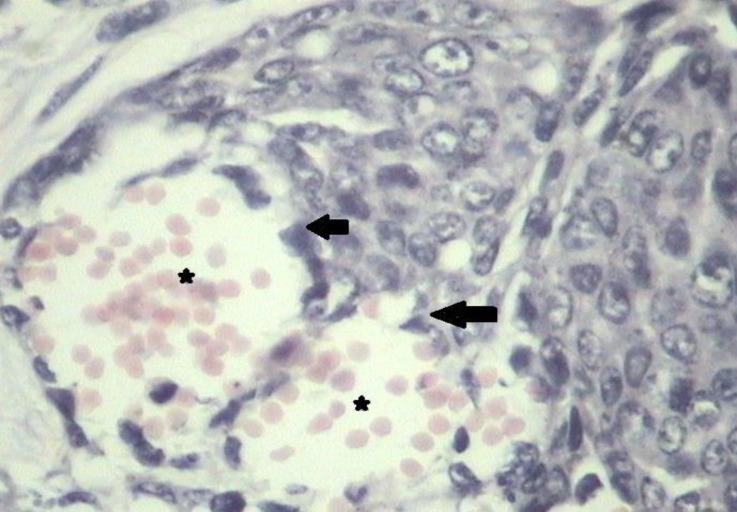
Carcinosarcoma invasion of neoplastic cells (arrows) to vessels (stars), (H & E, 400×).

**Fig. 5:**
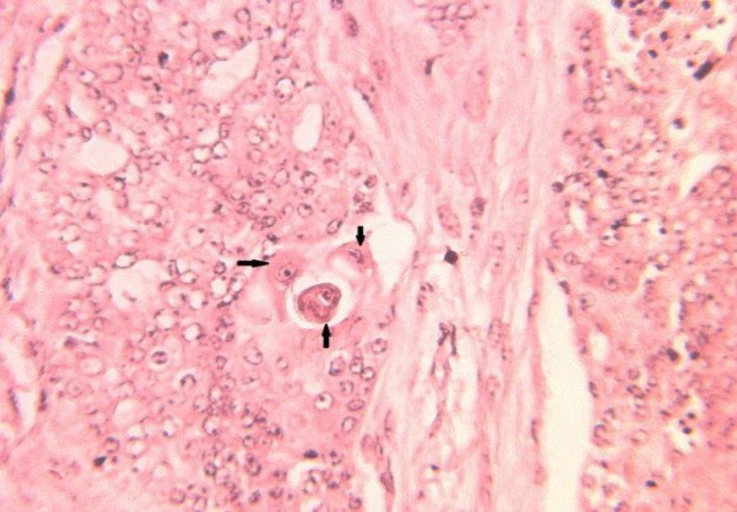
Carcinosarcoma. Note the bizarre cells (arrows), (H & E, 400×).

**Fig. 6:**
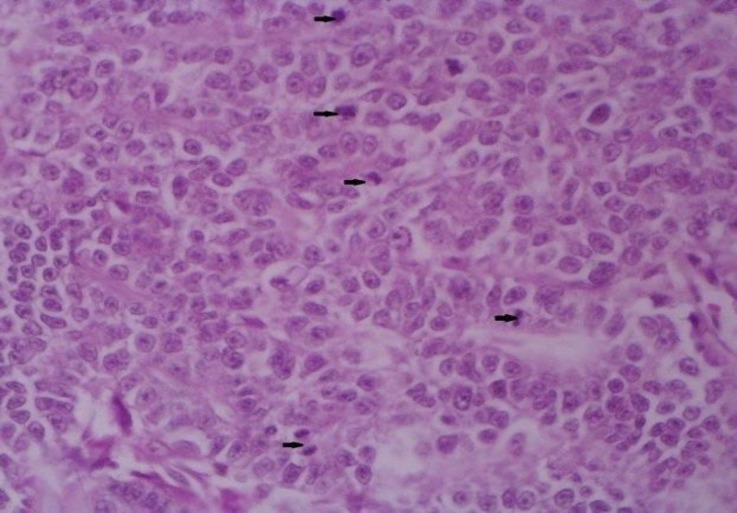
Note the high mitotic index and also different mitotic figures (arrows), (H & E, 400×).

**Table 3:** Relationship between histological grading and tumor type in 30 dogs with mammary carcinoma (%).

**Histopathological type**	**I**	**II**	**III**	**Total**
Simple carcinoma	10 (47.6)	9 (42.9)	2 (9.5)	21 (65.6)
Complex carcinoma	-	5 (100)	-	5 (15.6)
Carcinoma arising in benign tumor	2 (50.0)	1 (2.05)	1 (25.0)	4 (6.7)
Special type of carcinoma	-	-	2 (100)	2 (6.3)
Total	12 (37.5)	15 (46.9)	5 (15.6)	32 (100)

**Table 4:** Relationship between histological grading and sub classification of simple carcinoma in 21 bitches with mammary gland carcinoma (%).

**Histopathological type**	**I**	**II**	**III**	**Total**
Tubulopapillary carcinoma	10 (58.8)	7 (41.2)	-	17 (100)
Solid carcinoma	-	-	1 (100)	1 (100)
Cribriform carcinoma	-	2 (66.7)	1 (33.3)	3 (100)
Total	10 (47.6)	9 (42.9)	2 (9.5)	21 (100)

**Table 5:** Relationship between histological grading and sub classification of special type of carcinoma in 2 bitches with mammary gland carcinoma (%).

**Histopathological type**	**I**	**II**	**III**	**Total**
Lipid-rich tumor	-	-	1 (100)	1(100)
Spindle cell tumor	-	-	1 (100)	1(100)
Total	0	0	2 (100)	2 (100)

## Discussion

One of the major challenges for a veterinary oncologist is to identify the prognostic variables which allowed disease behavior to be predicted.^9^

In canine mammary neoplasm, tumor type was an important factor and an increasing range was observed in malignancy of complex carcinoma (composed of both epithelial and myo-epithelial components) to simple carcinoma (composed of the one type of cell - either epithelial or myo-epithelial like cells) to sarcoma.^6^

Some studies showed that half (42.0-55.0%) of the surgically removed mammary tumors in bitches were malignant.^16^ Meuten reported that about 20.0-40.0% of bitches with mammary tumors developed malignant kinds.^17^ Although Simeonov and Stoikov reported that only 19.0% benign and 81.0% mammary tumors were malignant.^18^ In the present study all samples (n = 37) were malignant. Rezaie *et al.* found that 70.6% of bitches had tubulopapillary carcinoma, 23.5%- solid carcinoma, and 5.9% - cribriform carcinoma.^15^ Ežerskytė *et al. *showed that the most common tumor types of mammary glands in bitches were simple carcinoma, complex carcinoma and carcinosarcoma 46.0%, 27.0% and 13.0%, respectively.^19^

In the present study, 86.5 % (n = 32), and 13.5 % (n = 5) of mammary tumors were carcinomas and sarcomas, respectively. The most frequently represented tumor type was simple carcinoma 56.8% (n = 21), followed by complex carcinoma 13.5% (n = 5), sarcoma 13.5% (n = 5), carcinoma arising from benign tumor 10.8% (n= 4) and special type of carcinoma 5.4% (n = 2).

In sub classification of simple carcinomas: 80.9% (n = 17) tubulopapillary carcinoma, 4.8% (n = 1) solid carcinoma and 14.3% (n = 3) cribriform carcinoma. These results were in agreement with above researches.

Most grading systems of mammary carcinomas in dogs are a modification of the numeric method of Elston and Ellis.^10^^,^^20^ The Elston and Ellis grading method has been used previously to study canine mammary carcinoma^10^^,^^15^^,^^21^^,^^22^ and feline mammary carcinomas.^23^^-^^25^

In human, the combination of histological type and grade produce a more accurate assessment of prognosis of breast cancer than histological type alone. Histological grade may also provide useful information to predict the response to chemotherapy and, therefore, be a predictive factor.^11^

In this study, most carcinomas were graded II and I, with just 15.6 % of lesions being classiﬁed as grade III and also we found complex carcinoma and carcinoma arising from benign mixed tumor were usually of grade I or II. The presence of myo-epithelial cells in complex carcinomas is associated with a less aggressive biological behavior and a better prognosis than with simple carcinomas.^2^^,^^6^ These results are in agreement with other studies.^10^^,^^15^

Ten samples out of 12 samples of grade I (83.3%) were tubulopapillary carcinoma, that were consisted of more than 75.0% tubules with a clear central lumina, cells with regular outline and uniform and also less than 7 mitosis per 10 HPF. On the other hands, simple solid carcinoma (more malignant than tubulopaillary) was grade III. Cribriform carcinoma, which is uncommon, is characterized by the proliferation of a population of neoplastic epithelial cells forming a sieve-like arrangement.^26^ In this study we described 3 simple cribriform carcinoma (two grade II and one grade III). This type of tumor has worse prognoses than tubulopapillary and solid carcinoma.

The malignant mixed mammary tumor (carcinosarcoma) is composed partly of cells morphologically resembling the epithelial component and partly of cells morphologically resembling connective tissue elements, both types of which are malignant.^27^^,^^28^ It is an uncommon mammary gland neoplasm, but it most often presents as a carcinoma and osteosarcoma. The epithelial component metastasizes via lymphatic vessels to regional lymph nodes and the lungs, and the mesenchymal component, via the hematogenous route to the lungs.^26^

Matrix-producing carcinoma is a very rare breast neoplasm accounting for less than 0.1% of all human breast malignancies.^29^ There are limited reports of canine primary mammary chondrosarcoma in the literature. Present paper described a primary mammary chondrosarcoma. According to authors’ knowledge, this is the first report of this kind of mammary tumor in Iran.

The prognosis is based on multiple factors. The type of tumor is important in determining the prognosis. Sarcomas are associated with shorter survival times than carcinomas. Other factors for poor prognosis are size of tumor, lymph node involvement, ulcerated tumor surface, rapid growth of tumor, tumor adherence to deeper tissues and nuclear differentiation.^30^

Karayannopoulou *et al. *found significant differences in survival between cases with different tumor grades.^10^ Survival was worse in dogs with grade III carcinomas than in those with grade I or grade II. Dogs with simple carcinomas had a worse prognosis than with other carcinomas; there was no significant difference in survival between grade II and grade III cases, with both having a very poor prognosis. Undifferentiated carcinomas (grade III) had an increased risk of death when compared with differentiated carcinomas (grade I and II). As noted in many of the studies, lymphatic/vascular invasion and lymph node metastasis are, as expected, associated with a poor prognosis.^26^ In feline mammary carcinoma there was a signiﬁcantly shorter overall survival in queens with grade III tumors, which was proved in other reports.^23^^,^^25^ In this study, cases graded III had significant invasion to blood and lymphatic vessels.

This study demonstrated that the Elston and Ellis method of histological grading in canine mammary tumor is a reliable prognostic factor. That is correlated with histopathological classification. Grading should be a usual procedure in the evaluation of biological aggressiveness of canine mammary carcinomas. Its routine use should be helpful in indicating appropriate post-surgical treatment.
